# Fasting plasma glucose and fetal ultrasound predict the occurrence of neonatal macrosomia in gestational diabetes mellitus

**DOI:** 10.1186/s12884-023-05594-6

**Published:** 2023-04-19

**Authors:** Yuting Zhang, Linying Chen, Lijing Zhang, Yudan Wu, Li Li

**Affiliations:** 1 The First Affiliated Hospital of Shantou University Medical College, Shantou, Guangdong China; 2 Shantou University Medical College, Shantou, Guangdong China

**Keywords:** Gestational diabetes mellitus, Prediction, Fasting plasma glucose, Fetal ultrasound, Neonatal macrosomia

## Abstract

**Objective:**

The cause of fetal overgrowth during pregnancy is still unclear. This study aimed to analyze and predict the risk of macrosomia in pregnant women with gestational diabetes mellitus (GDM).

**Methods:**

This study was a retrospective study collected from October 2020 to October 2021. A total of 6072 pregnant women with a routine 75-g oral glucose tolerance test (OGTT) during 24–28 gestational weeks were screened. Nearly equal numbers of pregnant women with gestational diabetes and with normal glucose tolerance (NGT) were included in the study. Multivariate logistic regression analysis and receiver operating characteristic (ROC) curve were performed to determine the index and inflection point for predicting macrosomia occurrence.

**Results:**

The data of perinatal outcomes of 322 GDM and 353 NGT who had given birth to single live babies at term were analyzed. We found that significant cut-off values for the prediction of macrosomia are 5.13mmol/L in fasting plasma glucose (FPG), 12.25kg in gestational weight gain (GWG), 3,605g in ultrasound fetal weight gain (FWG) and 124mm in amniotic fluid index (AFI).The area under the ROC curve of this predictive model combined all variables reached 0.953 (95% CI: 0.914 ~ 0.993) with a sensitivity of 95.0% and a specificity of 85.4%.

**Conclusions:**

FPG is positively associated with newborn birth weight. An early intervention to prevent macrosomia may be possible by combining maternal GWG, FPG, FWG, and AFI in gestational diabetes.

## Background

During pregnancy, gestational diabetes mellitus(GDM) is the most common condition associated with hyperglycemia. International prevalence of GDM continues to rise due to epidemiological factors, including obesity rates among women of reproductive age, maternal age increases, and revisions to GDM criteria and diagnostic procedures from the International Association of Diabetes and Pregnancy Study Groups (IADPSG) [1, 2]. Intrauterine hyperglycemia environment can affect a pregnant woman’s metabolism, immune system, and reproductive health. There are some evidences that GDM is associated with polycystic ovary syndrome (PCOS), obesity, and hyperinsulinemia later in life and is a risk factor for cardiometabolic diseases in the mother and her offspring [3]. It is known that gestational diabetes mellitus can cause obstetric and neonatal complications, particularly increasing birth weight in infants [4, 5]. Macrosomia is a common adverse outcome of gestational diabetes. In neonates, a high birth weight increases the risk of shoulder dystocia and birth injury (clavicular fracture or brachial plexus injury) which cause the major neonatal admission to the intensive care unit. In the long run, macrosomia has a significantly higher risk not only of developing overweight or obesity in adulthood, but also of developing type 2 diabetes, hypertension, and cardiovascular disease [4–7].

Prediction of macrosomia remains a challenge. Macrosomia is more common in women with gestational diabetes or pre-pregnancy diabetes than in women without diabetes, which may sometimes be associated with maternal glycemic control [8]. A recent study investigating glucose levels in pregnant women with GDM in the first, second, and third trimesters found that maternal hyperglycemia is associated with increased fetal growth, large gestational age infants (LGA), and macrosomia before the diagnosis of GDM [9]. The Deniz Esinler et al. Study found that fetal abdominal circumference predicts large birth weights better than other fetal ultrasound soft indicators. However, studies have yet to find a weighted formula that can accurately predict macrosomia [10].

As of yet, the influencing factors and prediction methods of macrosomia remain unclear. Consequently, this study aimed to investigate the predictive power of fasting blood glucose and other factors for macrosomia by analyzing different influencing factors of macrosomia.

## Methods

### Research objects

Our study conducted a retrospective study from October 2020 to October 2021 including pregnant women aged 20–45 who gave birth at the Obstetrics Medical Center of the First Affiliated Hospital of Shantou University Medical College which was a local tertiary health care center. Accepting the odds ratio of macrosomia incidence in the case group compared to the control group of about 1.5 and α of 0.05, we required a sample size of at least 155 individuals in each group to achieve 80% power.

According to the epidemiological standard, we conducted questionnaire surveys to screen pregnant women who lived in Shantou City for more than 5 years, had a mainly carbohydrate-based diet, aged form 20 to 45 years old and had a gestational age greater than or equal to 37 weeks. Through questionnaire surveys, there were 6072 voluntary pregnant women recruited in the study. In the first round of screening, pregnant women with multiple pregnancies; with uncertain last menstrual period data or gestational age less than 37w; with age older than 45y or younger than 18y; with miscarriages, stillbirths or terminators; with previous history of preexisting major chronic conditions or diabetes; with LMP date or gestational age less than 37w or with using of insulin for abnormal blood glucose were excluded. There were 2839 pregnant women and offspring left after the first round of screening. In the second round of screening, pregnant women with missing OGTT test results; with missing FPG data; with no ultrasound data or with duplicate or invalid values were excluded. Finally, we included 322 GDM pregnant women and 353 non-GDM pregnant women brought full-term single-live babies to our hospital.

By the IADPSG standard, the GDM samples are further divided into five groups:

1. No abnormal glucose tolerance (non-GDM): FPG < 5.1mmol/L, 1hPG < 10.0mmol/L and 2hPG < 8.5 mmol /L.

2. Impaired fasting blood glucose (i-IFG): FPG ≥ 5.1mmol/L ,1hPG < 10.0mmol/L and 2hPG < 8.5mmol/L.

3. Single isolated impaired glucose tolerance (i-IGT1): FPG < 5.1mmol/L, 1hPG ≥ 10.0mmol/L or 2hPG ≥ 8.5 mmol/L.

4. Double impaired glucose tolerance (i-IGT2): FPG < 5.1mmol/, 1hPG ≥ 10.0mmol/L and 2hPG ≥ 8.5mmol/L.

5. Combined impaired fasting glucose and glucose tolerance (IFG/IGT): FPG ≥ 5.1mmol/L, 1hPG ≥ 10.0mmol/L and 2hPG ≥ 8.5mmol/L.

**Fig. 1:**
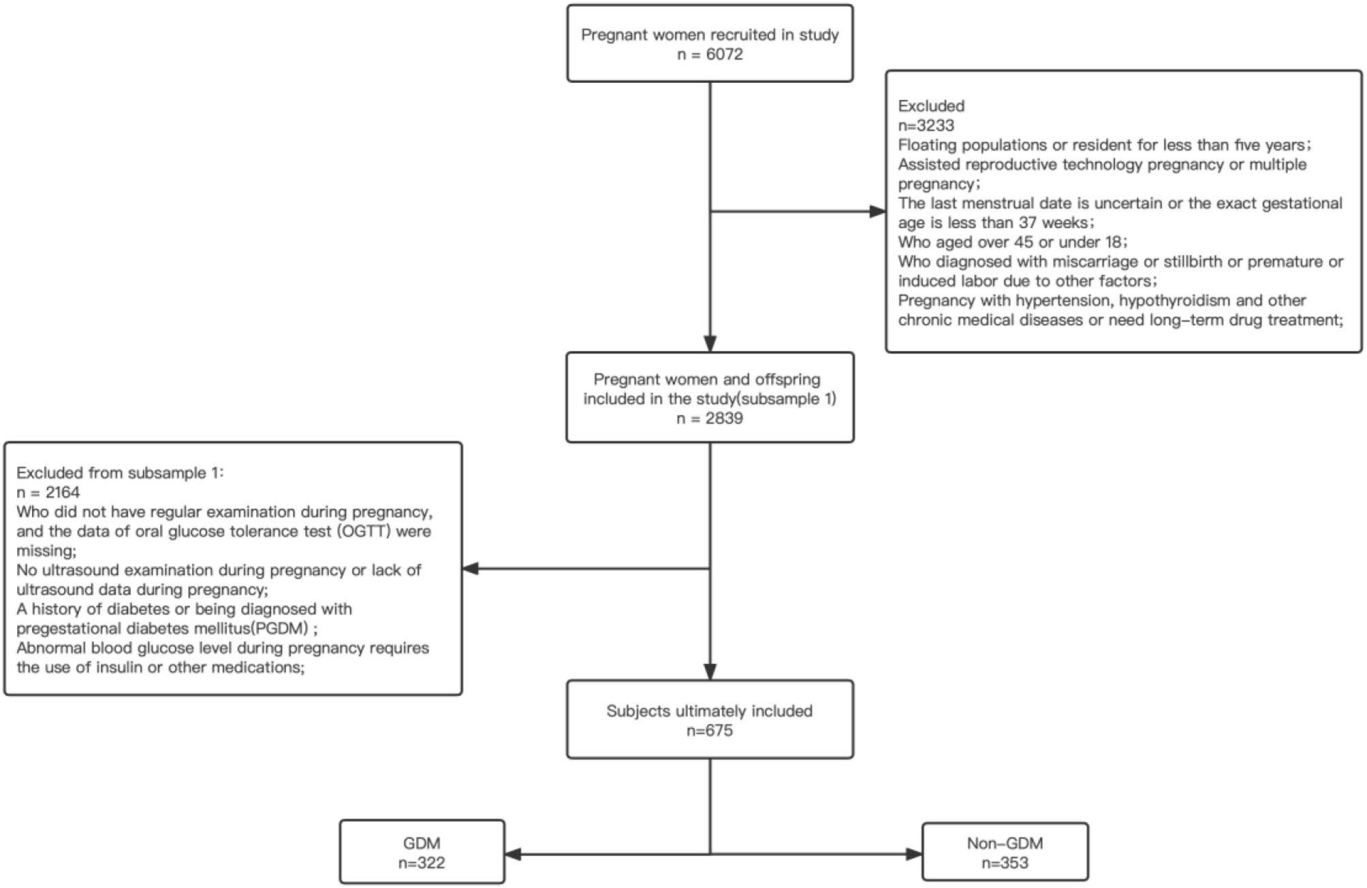
Variable screening flow chart

The process of selecting participants is shown in Fig. 1. All study participants provided informed consent.The study was approved by Ethics Committee of Shantou University Medical College. The number of ethic approval is 2020 − 109.

### Research methods

Our research specialists performed a one-step 75-gram oral glucose tolerance test (OGTT) on all subjects between 24 and 28 weeks of gestation. GDM was diagnosed by the IADPSG criteria when any of the following criteria was met on the 75-g OGTT: fasting plasma glucose (FPG) ≥ 5.1mmol/L; 1-h value (1 h-PG) ≥ 10.0 mmol/L; and 2-h value (2 h-PG) ≥ 8.5 mmol/L [11, 12]. The subjects should fast for 10–16 h before the test. The commissioner took 2ml of venous blood from the subjects and put it in the vacuum coagulation tube, which was detected by an automatic biochemical analyzer, model: CX5DELTA; manufacturer: Beckman company; reagent: produced by Shanghai Kehua Bioengineering; detection method: glucose oxidase method. All participants with GDM were asked to self-monitoring of blood glucose (SMBG) profiles (including fasting and 2-h after breakfast, lunch, and dinner, at least three times a week) and a diet diary every day. We asked for the total caloric intake to be 30–35 kcal/kg body weight for average women, 25–30 kcal/kg desirable for overweight/obese women. During their hospitalizations for delivery, we collected general information about these women, including age, pre-pregnancy weight, parity, mode of delivery, gestational age at delivery, family history of diabetes, last menstrual period, pre-delivery weight, and OGTT FPG, 1hPG, and 2hPG measurements, perform routine blood collection, coagulation testing, liver and kidney functions, blood lipid testing, etc. A fetal ultrasound was performed in all of the study participants within the 72-h period prior to the vaginal/ cesarean delivery, using a Voluson E6 ultrasound device (GE Healthcare, Chicago, USA) equipped with 1–5 MHz convex transducer. All ultrasound measurements were performed by trained sonographers or obstetric specialists. We collected prenatal fetal ultrasound measurements of pregnant women, including ultrasound estimated fetal weight (EFW), long bone length (femoral length/humeral length (FL/HL)), head circumference (HC), abdominal circumference (AC), etc. Pre-pregnancy body mass index (BMI) was calculated by dividing weight (kg) by the square of height (m^2^).Maternal gestational weight gain (GWG) was calculated as pre-delivery weight minus pre-pregnancy weight. After these women gave birth, we collected information on the newborn, including Apgar score at birth, birth weight, postnatal blood glucose, and transcutaneous bilirubin measurement. Macrosomia was defined as full-term birth weight ≥ 4,000g.

### Statistical analysis

Data were analyzed utilizing SPSS 26.0. Using the Shapiro-Wilk (SW) method to test the normality of variables. ANOVA one-way analysis of normality variance was used to compare the means of variables between multiple groups that fit the normal distribution. The proportion of outcome variables was analyzed using χ2-test. Spearman’s correlation analysis was used to find out the correlation, and logistic regression analysis was conducted to determine the risk factors of GDM. According to the regression analysis results, a risk prediction model was established, and the receiver operating characteristic (ROC) curve was calculated to evaluate the predictive value of the regression model. The accuracy of the models was evaluated using the area under the curve (AUC) with 95% confidence interval (CI). A *p* value less than 0.05 was considered as statistically significant.

Based on the p-value (p ≤ 0.1) of univariate logistic regression and literature review, we adjusted for potential confounding factors associated with macrosomia, including gestational age, gestational age, pre-pregnancy BMI, family history of diabetes, ultrasound estimated fetal double top diameter (BPD), head circumference (HC), abdominal circumference (AC), femoral length (FL) and humeral length (HL).

## Results

a) **Based on the IADPSG groupings, a comparison of general conditions among pregnant women was made.**

**Fig. 2:**
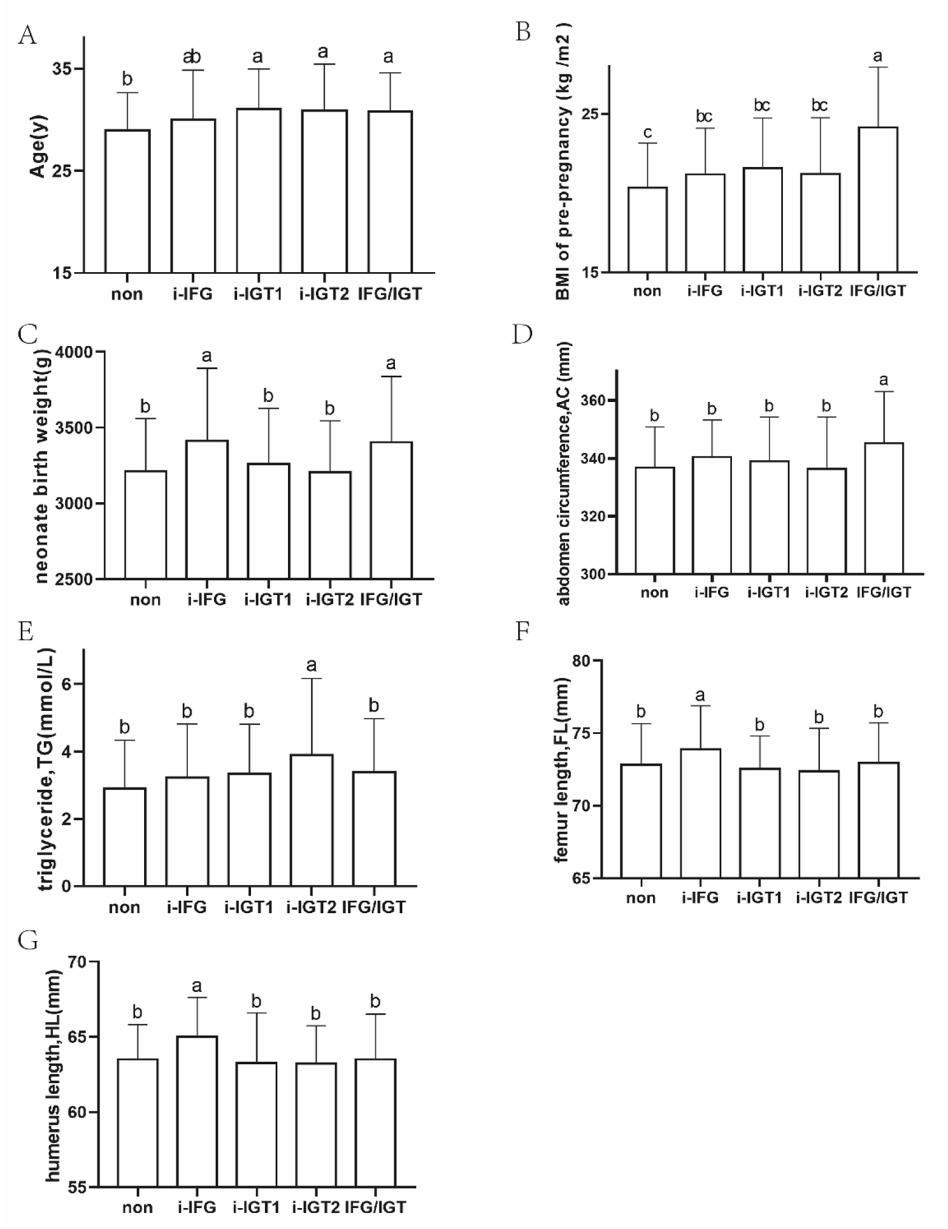
Based on the expression level of each factor under IADPSG grouping. **A**: Pregnant Age (y) **B**: BMI of pre-pregnancy (kg /m^2^) **C**: neonate birth weight (g) **D**: ultrasound estimating fetal abdominal circumference, AC **E**: Maternal blood triglycerides, TG **F**: ultrasound estimating fetal femur length, FL **G**: ultrasound estimating fetal humerus length, HL

The IADPSG diagnostic criteria identified 322 patients with GDM (GDM group), including 59 in the IFG group, 143 in the i-IGT1, 61 in the i-IGT2, 59 in the IFG/IGT group, and 353 patients without GDM (non-GDM group). The age of the pregnant in i-IGT1 was the highest (31.04 ± 3.93y). However, the post-hoc comparison and multiple analysis found that only the nongroup and four other groups were statistically significant, i.e., no significant differences between the four groups of GDM pregnant women, as shown in Fig. 2. Pregnant women’s pre-pregnancy weight (60.97 ± 10.78kg) and pre-pregnancy BMI (24.13 ± 3.82kg/m^2^) were higher in the IFG/IGT group. GWG (13.89 ± 4.34kg) was higher in the non-GDM group, which may be correlated with our diet control results in GDM pregnant women. Pregnant women’s prenatal ultrasound amniotic fluid index (AFI) was the highest (108.18 ± 37.56mm) in the i-IGT2 group. Triglycerides (TG), Low-density lipoproteins (LDL), and Total cholesterol (TC) were all higher in the i-IGT2 group (3.89 ± 2.29mmol/L,4.07 ± 0.82mmol/L,7.27 ± 1.44mmol/L). Results shown in Table 1.

**Table 1:** Comparison of general conditions of pregnant women in each group based on IADPSG grouping (x ± s)

Characteristics	GDM subtypes according to IADPSG thresholds Groups					F	P
	non (n = 353)	i-IFG (n = 59)	i-IGT1 (n = 143)	i-IGT2 (n = 61)	IFG/IGT (n = 59)		
Age(y)	28.92 ± 3.7	30.00 ± 4.84	31.04 ± 3.93	30.90 ± 4.53	30.80 ± 3.78	10.069	< 0.001
gestational weeks(week)	38.89 ± 1.02	38.78 ± 1.16	38.63 ± 0.87	38.57 ± 0.92	38.78 ± 0.89	2.639	0.033
Weight of pre-pregnancy (kg)	51.53 ± 7.73	54.15 ± 7.87	54.04 ± 8.48	52.96 ± 9.68	60.97 ± 10.78	16.464	< 0.001
BMI of pre-pregnancy (kg /m^2^)	20.34 ± 2.81	21.17 ± 2.92	21.57 ± 3.16	21.21 ± 3.55	24.13 ± 3.82	20.478	< 0.001
gestational weight gain, GWG (kg)	13.89 ± 4.34	13.69 ± 3.92	12.36 ± 3.85	11.98 ± 4.47	12.29 ± 4.95	5.772	< 0.001
Amniotic fluid index, AFI(mm)	94.36 ± 33.53	95.54 ± 42.28	93.51 ± 31.5	108.18 ± 37.56	98.84 ± 33.24	2.415	0.048
Amniotic Fluid vertical depth, AFV(mm)	37.55 ± 11.38	36.82 ± 12.47	38.06 ± 11.35	41.31 ± 12.13	38.66 ± 12.16	1.570	0.181
red blood cell count, RBC (×10^12^/L)	4.00 ± 0.39	3.97 ± 0.33	4.05 ± 0.39	4.12 ± 0.36	4.17 ± 0.4	3.429	0.109
white blood cell count, WBC (×10^12^/L)	10.16 ± 3.05	9.18 ± 2.49	9.34 ± 2.71	8.55 ± 2.35	8.64 ± 2.14	7.989	0.101
blood platelet count, PLT (×10^12^/L)	227.10 ± 52.31	219.41 ± 50.84	221.08 ± 58.68	220.05 ± 65.78	227.09 ± 63.17	0.573	0.682
D_Dimer	2724.73 ± 1822.88	2526.61 ± 1677.88	2887.99 ± 2193.45	2669.67 ± 1446.53	2446.42 ± 1317.18	0.798	0.526
Alamine aminotransferase, ALT (U/L)	11.34 ± 5.44	11.96 ± 9.08	13.52 ± 21.27	18.51 ± 37.25	11.73 ± 6.55	2.896	0.420
aspartate aminotransferase, AST(U/L)	17.77 ± 4.7	17.63 ± 6.49	18.36 ± 9.21	21.39 ± 18.99	17.35 ± 5.45	2.736	0.544
serum creatinine,SCr (µmol/L)	57.67 ± 9.18	57.85 ± 10.35	57.78 ± 9.56	56.32 ± 9.08	57.12 ± 9.05	0.338	0.853
triglyceride, TG (mmol/L)	2.90 ± 1.44	3.23 ± 1.59	3.33 ± 1.47	3.89 ± 2.29	3.38 ± 1.60	6.236	0.001
low-density lipoprotein, LDL (mmol/L)	3.82 ± 0.82	3.82 ± 0.87	3.66 ± 0.81	4.07 ± 0.82	3.50 ± 0.77	4.442	0.002
total cholesterol, TC (mmol/L)	6.67 ± 1.31	6.70 ± 1.28	6.60 ± 1.25	7.27 ± 1.44	6.27 ± 1.19	4.586	0.001

Neonatal weight (3413.73 ± 478.95g) and ultrasound measurements of FL (73.89 ± 2.99mm) and HL (65.02 ± 2.62mm) were the highest in the i-IFG group. Ultrasound-estimated fetal weight (EFW) (3410.05 ± 431.97g) and AC (345.14 ± 17.98mm) were the highest in the IFG/IGT group. Macrosomia incidence was highest among i-IFG participants (16.9%). Table 2 shows the results. To compare intra-group differences further, we selected several variables with high specificity (Fig. 2).

**Table 2:** Comparison of newborns in each group based on IADPSG grouping (x ± s)/(n (%))

Characteristics	GDM subtypes according to IADPSG thresholds Groups					F	P
	non	i-IFG	i-IGT1	i-IGT2	IFG/IGT		
n	351	59	142	61	59		
neonate birth weight (g)	3212.62 ± 348.41	3413.73 ± 478.95	3262.24 ± 366.5	3205.36 ± 340.01	3403.78 ± 433.83	6.445	0.001
ultrasound estimating fetal weight, EFW (g)	3258.77 ± 323.76	3361.79 ± 322.33	3294.04 ± 319.64	3232.34 ± 378.89	3410.05 ± 431.97	3.62	0.006
pulsed index, PI	0.84 ± 0.21	0.88 ± 0.24	0.81 ± 0.16	0.86 ± 0.31	0.85 ± 0.3	1.165	0.325
resistance index, RI	0.57 ± 0.3	0.55 ± 0.06	0.55 ± 0.07	0.55 ± 0.07	0.56 ± 0.15	0.237	0.918
Umbilical artery blood flow velocity peak to valley ratio, S/D	2.24 ± 0.37	2.26 ± 0.35	2.21 ± 0.33	2.25 ± 0.35	2.23 ± 0.35	0.305	0.875
biparietal diameter, BPD (mm)	92.79 ± 3.05	93.11 ± 3.12	92.81 ± 3.22	92.05 ± 3.19	92.98 ± 4.02	1.006	0.404
head circumference, HC (mm)	330.43 ± 11.12	332.53 ± 8.61	329.69 ± 18.58	330.61 ± 10.51	332.9 ± 10.4	0.974	0.421
abdomen circumference, AC (mm)	336.65 ± 14.17	340.33 ± 12.88	339.01 ± 15.24	336.31 ± 17.89	345.14 ± 17.98	4.637	0.001
femur length, FL (mm)	72.84 ± 2.83	73.89 ± 2.99	72.53 ± 2.29	72.34 ± 2.98	72.97 ± 2.74	3.088	0.016
humerus length, HL (mm)	63.5 ± 2.32	65.02 ± 2.62	63.25 ± 3.35	63.25 ± 2.49	63.5 ± 3.02	4.961	0.001
macrosomia(n(%))	7(2.0)	10(16.9)	3(2.1)	1(1.6)	7(11.9)	/	< 0.001

b) **The influence of gestational diabetes mellitus on macrosomia in pregnant women**.

**Fig. 3:**
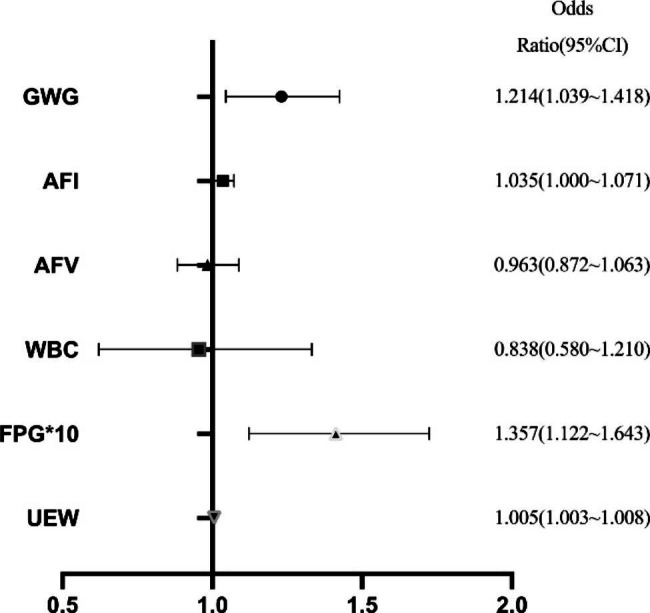
Variables affecting the incidence of macrosomia. CI, confidence interval; UEW, Ultrasound estimation of fetal weight; GWG, gestational weight gain

Based on the diagnosis outcome, pregnant women with GDM were divided into macrosomia and non-macrosomia groups. In the first step, we conducted a univariate analysis of the variables and screened out any independent variables correlated with macrosomia (GWG, FPG, AFI, AFV, WBC, EFW, BPD, HC, AC, FL, HL). The variables derived from correlation analysis were used as independent variables in a multivariate logistic regression analysis with macrosomia as the dependent variable. Regression analysis results suggest that GWG, FPG, AFI, EFW, and ultrasound measurement of fetal AC and HL were the influencing factors of macrosomia. After excluding variable collinearity, multivariate regression analysis was carried out on the included variables. The results showed GWG, FPG, AFI, and EFW were the influencing factors for the occurrence of macrosomia. Every 1 kg of weight gain during pregnancy increases the risk of macrosomia by 1.221 times (95%CI:1.045–1.425). An increase in FPG of 0.1mmol/L increases macrosomia risk by 1.391 times (95%CI:1.122 ~ 1.724). Figure 3 illustrates the detailed results of the multivariate logistic regression analysis.

c) **ROC curve analysis for macrosomia prediction**.

**Fig. 4:**
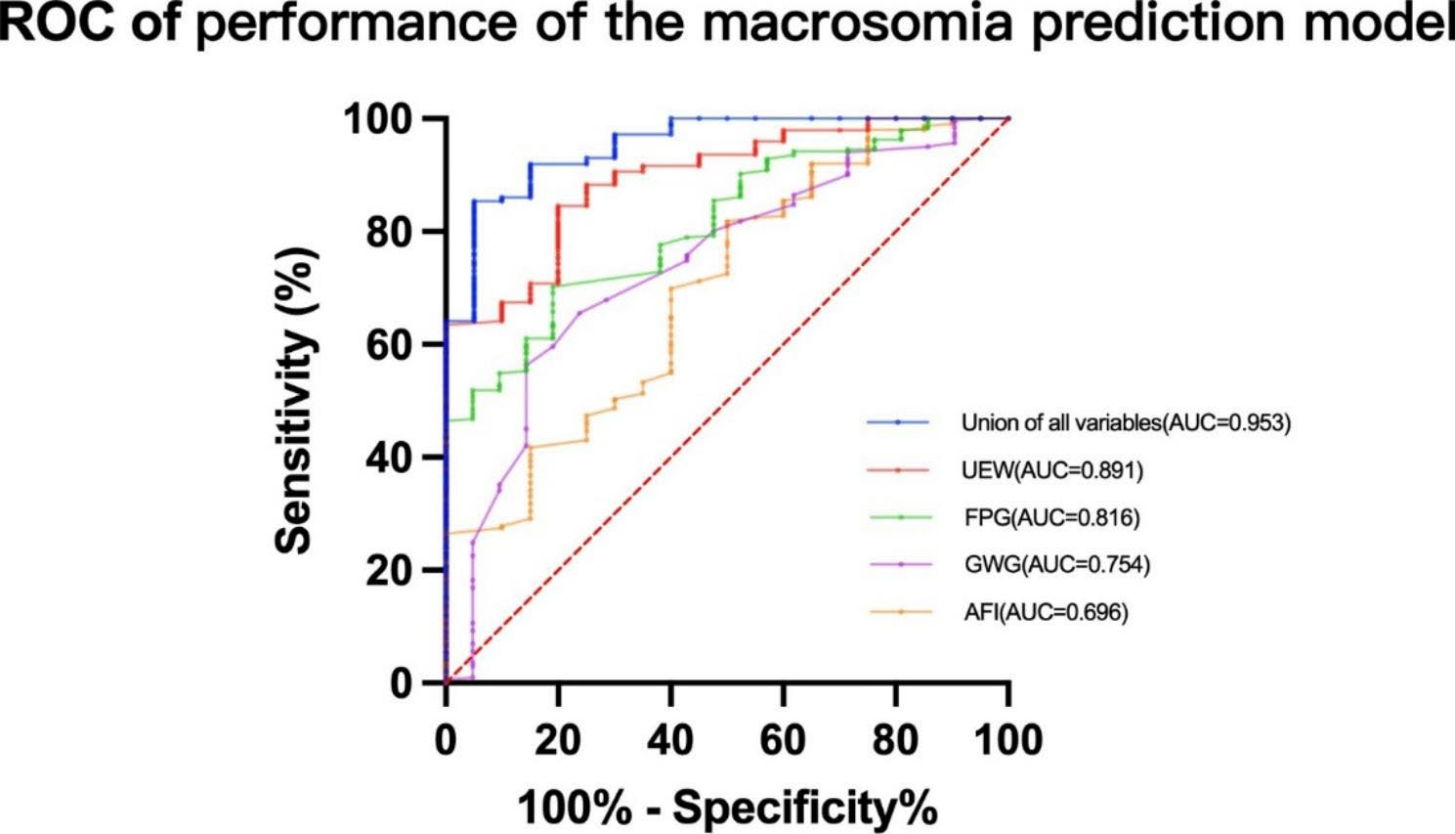
ROC curve analysis of the performance of the macrosomia prediction model. AUC, area under curve; UEW, Ultrasound estimation of fetal weight; GWG, gestational weight gain

GWG, AFI, FPG, and EFW were macrosomia risk factors after excluding collinear interference. We analyzed the combined predictive power of all the above variables for macrosomia using ROC curve analysis. The area under the ROC curve of FPG reached 0.816 (95% CI: 0.735 ~ 0.897) with a sensitivity of 80.0% and a specificity of 70.4%. The cut-off value of GWG was 12.25kg; the cut-off value of AFI was 124mm; the cut-off value of FEW was 3,605g. As shown in Table 3, the area under the ROC curve of all variables reached 0.953 (95% CI: 0.914 ~ 0.993), with a sensitivity of 95.0% and a specificity of 85.4%. Figure 4 shows the ROC curve drawn.

**Table 3:** ROC curve analysis for risk prediction of macrosomia

Characteristics	AUC (95% CI)	Sensitivity (%)	Specificity (%)	Youden index	Cut-off point
Pregnancy gain weight(kg)	0.754(0.645 ~ 0.863)	90.00	56.10	46.10	12.25
Amniotic fluid index, AFI (mm)	0.696(0.577 ~ 0.815)	50.00	81.50	31.50	124
Oral Glucose Tolerance Test 0 h PG*10(mmol/L)	0.816(0.735 ~ 0.897)	80.00	70.40	50.40	5.125
Fetal weight estimated by ultrasound(g)	0.891(0.831 ~ 0.951)	80.00	84.00	64.00	3605
Union of all variables	0.953(0.914 ~ 0.993)	95.00	85.40	80.40	/

## Discussion

There were 675 cases in this study, and 322 pregnant women were diagnosed with GDM. Of those diagnosed with GDM, 18.32% had single impaired fasting blood glucose. Researchers have found that higher neonatal birth weight is closely related to abnormally elevated fasting blood glucose. In addition, high fasting glucose is also an independent risk factor for macrosomia. Further evidence indicates that fasting blood glucose can positively influence neonatal birth weight. GDM is characterized by relative insulin deficiency. Insulin secretion from maternal islet beta cells cannot compensate for the gradual increase in insulin resistance during pregnancy [13]. Pedersen et al. have also shown that maternal hyperglycemia causes hyperglycemia and hyperinsulinemia in the fetal circulation, resulting in diabetes in the fetus [14]. It is obvious that macrosomia in pregnancy is a metabolic reaction to hyperinsulinemia [15]. As a result of maternal insulin resistance,the levels of maternal postprandial glucose and free fatty acid are elevated, while placental pro-proliferation increases glucose availability for fetal development [16, 17]. Maternal hyperglycemia results in fetal hyperglycemia via the facilitated diffusion of glucose-by-glucose transporter 1 [18]. Fetal hyperglycemia results in fetal hyperinsulinemia, promoting fetal anabolism, excessive fetal adiposity, and accelerated growth, leading to LGA and macrosomia [19, 20]. According to several studies, the fetus will overgrow due to insulin metabolism and other factors [15, 21, 22]. Therefore, fasting blood glucose testing at the first prenatal visit can not only determine whether it exceeds the appropriate critical value (5.13mmol/L) for predicting macrosomia risk, it also may help identify occult GDM, type 2 diabetes, etc.

Additionally, the study found that indicators of fetal ultrasound measurements before delivery, including ultrasound estimated weight (EFW) and amniotic fluid index (AFI), can effectively predict macrosomia. Alexandros A. Moraitis et al. also found that EFW and AC were highly sensitive to macrosomia prediction [23]. According to consistency analysis (paired-sample t-test) (P = 0.01), the fetal weight estimated by EFW differed statistically from the actual birth weight of the newborn. Our solution to this problem was to determine the optimal cut-off value (3,605 g) for the prediction of macrosomia by ultrasound-estimated fetal weight through the ROC curve and then to divide the samples according to this value into “ultrasonic diagnosis of macrosomia” and “ultrasonic diagnosis of non-macrosomia”. Then, ultrasound diagnosis of macrosomia and actual macrosomia incidence were analyzed with chi-square tests. There was a statistically significant difference between the two groups in the accuracy of diagnosing macrosomia (P < 0.001). The reason why for this phenomenon may also be that although ultrasound measurement of weight can predict neonatal birth weight to some extent (there is a correlation between the two variables), ultrasound measurement cannot be used to estimate fetal weight in cases of macrosomia (birth weight exceeding 4,000 g). The narrow intrauterine space may result from the fetus’ excessive growth and development, which limits the ultrasound measurement angle. Several studies have shown that ultrasound measurements tend to overestimate the weight of tiny newborns and underestimate the weight of older infants and diabetic infants, particularly large infants with poor reliability [24]. According to N. J. Dudley’s study, ultrasound EFW is subject to a high degree of intra- and inter-observer variability, so formulas for estimating fetal weight need further improvement [25]. Historically, many studies have routinely used two-dimensional ultrasound to evaluate non-standard biometric parameters of fetuses in normal obstetrics, such as soft tissue thickness in the abdomen, humerus, mid-thigh, and cardiac morphology. Within China, abdominal circumference, head circumference, and long bone length are also commonly measured [25–29]. Mohamed et al. demonstrated that fetal abdominal fat layer thickness indicates macrosomia at 34 and 37 weeks of pregnancy [30]. In contrast to our study, he measured only the fetus’ abdominal wall fat thickness, which is different from the fetus’ abdominal circumference measured by our ultrasound. Despite the absence of interference from internal fetal organs, it cannot be excluded that long fetal bones, head circumference, etc., in large children could have influenced outcomes.

The i-IGT2 group had the highest TG, LDL, and TC levels among the five groups, consistent with Yuan Li et al.‘s findings [31]. This suggests that dual impaired glucose tolerance in pregnant women with GDM is more likely related to high blood lipid levels rather than to fetal growth or weight. There has been evidence that maternal hyperglycemia, significantly elevating OGTT 1hPG, may further reduce the function of placental cytotrophoblasts (CTB), and their secretion may become abnormal under hyperglycemia. This is responsible for decreased maternal glucose utilization, increased lipolysis, and increased lipid accumulation [32]. Furthermore, studies have shown that maternal blood triglyceride concentration is an independent predictor of large-for-gestational-age babies [33]. It differs from what we observed in our experiments. As our study used macrosomia as an adverse outcome instead of large-for-gestational-age (LGA), we found a positive correlation between triglyceride concentrations and neonatal weight. Still, abnormal maternal blood lipids may not be enough to diagnose macrosomia whose neonatal weight exceeds 4,000 g. Women from Chaoshan, Guangdong, are generally thin and small, which is also reflected in our data. The pre-pregnancy BMI is in the normal range or even low (BMI between 20.0 and 22.0 kg/m^2^), so we might not have been able to find an association between blood lipids and macrosomia. Further research is needed to understand the relationship between maternal blood lipids and postprandial blood glucose and the impact on adverse maternal and infant outcomes such as macrosomia.

Before childbirth, the sum of uterine height and abdominal circumference of pregnant women taller than or equal to 140 cm as a risk prediction for macrosomia. However, some pregnant women have thick belly wall fat. Data shows that Chinese women of childbearing age are more likely to have a BMI exceeding 25 kg/m^2^ [34]. Compared with normal-weight women, obese women are twice as likely to have a baby diagnosed with macrosomia [35]. Experience, however, indicates that obese women are more likely than normal-weight women to be misdiagnosed with macrosomia, especially when combined with GDM. Our study can add an effective prediction model or provide a clinical basis to macrosomia prediction. The measurement of FPG at the first prenatal visit may be critical in screening for previously undiagnosed preexisting diabetes [36]. We should learn how to screen women at the first prenatal visit for previously unknown diabetes or predict adverse outcomes for mothers and their babies, such as macrosomia, in advance.

In this study, we concluded that we should focus on the fetal ultrasound of pregnant women with high-risk factors (weight gain during pregnancy over 12.5 kg or FPG over 5.13mmol/L) in the third trimester; if the AFI is taller than 124 mm, estimated weight is more elevated than 3,605 g, special attention should be paid to the incidence of macrosomia. We identified predictors of macrosomia and their optimal cutoff values in this study, which will help clinicians better understand the possibility of GDM pregnant women developing macrosomia. Then they can choose to terminate the pregnancy early, reducing passiveness and preventing more unhealthy mothers from becoming pregnant. Clinical data from this study support the doctor’s recommendation that “pregnant women diagnosed with GDM through abnormal fasting blood glucose should strengthen weight and diet management during pregnancy, correct lifestyle education, and strengthen follow-up in the third trimester “[37].

Several limitations were found in this study. First, our pre-delivery ultrasound results were obtained from our hospital, which may limit the accuracy and objectivity of the statistical significance of the results. The formula used to calculate fetal ultrasound estimated weight is limited and cannot accurately represent all ultrasound weight estimates. However, it is also possible to exclude data errors due to different hospital ultrasound machines and measurement angles. Second, previous study indicated that the potential role of first trimester fetal heart rate and second trimester liver length in the early prediction of GDM [37–41]. In our study, although we focused on the predictors in third trimester, in the further study, some early predictors in first/second trimester could be contained. Moreover, since most of the data were collected when pregnant women provided their medical histories after admission, there would inevitably be some human error in their pre-pregnancy weight, pre-pregnancy BMI, and pregnancy weight gain. Therefore, our research employs professional researchers to collect and record patient information uniformly and to try to record the patient’s weight data at the first check-up. In addition, the sample data we included are all Asian populations from Guangdong, China, so our research results cannot represent the overall situation of all populations in the world.

## Conclusion

In conclusion, this study indicated that abnormally elevated FPG is an independent risk factor for macrosomia in pregnant women with GDM. In addition, when macrosomia is possible but cannot be diagnosed, a combination of maternal GWG, FPG, FWG, and AFI can predict macrosomia in gestational diabetes mellitus, which might be a new target for early intervention to prevent macrosomia.

## Data Availability

The raw data supporting the conclusions of this article will be made available by the authors, without under reservation. The datasets used and/or analysed during the current study available from the corresponding author on reasonable request.
